# The Hydrogenation Impact on Electronic Properties of p-Diamond/n-Si Heterojunctions

**DOI:** 10.3390/ma14216615

**Published:** 2021-11-03

**Authors:** Szymon Łoś, Kazimierz Fabisiak, Kazimierz Paprocki, Mirosław Szybowicz, Anna Dychalska, Ewa Spychaj-Fabisiak, Wojciech Franków

**Affiliations:** 1 Institute of Mathematics and Physics, Bydgoszcz University of Science and Technology, Profesora Sylwestra Kaliskiego 7, 85796 Bydgoszcz, Poland; 2 Department of Production Engineering Management, University of Bydgoszcz, Unii Lubelskiej 4c, 85059 Bydgoszcz, Poland; 3 Institute of Physics, Kazimierz Wielki University, Jana Karola Chodkiewicza 3, 85064 Bydgoszcz, Poland; paprocki@ukw.edu.pl (K.P.); franek3182@wp.pl (W.F.); 4 Faculty of Materials Engineering and Technical Physics, Institute of Materials Research and Quantum Engineering, Poznań University of Technology, Piotrowo 3, 61138 Poznań, Poland; miroslaw.szybowicz@put.poznan.pl (M.S.); anna.dychalska@put.poznan.pl (A.D.); 5 Department of Agricultural Chemistry, Faculty of Agriculture and Biotechnology, Bydgoszcz University of Science and Technology, Seminaryjna 5, 85326 Bydgoszcz, Poland; ewa.spychaj-fabisiak@pbs.edu.pl

**Keywords:** CVD diamond, Raman spectroscopy, p-diamond/n-Si heterojunction, SEM, J-V/T characteristic, hydrogenation

## Abstract

The undoped polycrystalline diamond films (PDFs) have been deposited on n-type silicon (Si) by Hot Filament Chemical Vapor Deposition (HF CVD) technique. The reaction gases are a mixture of methane and hydrogen. The obtained PDFs were characterized by scanning electron microscopy (SEM) and Raman spectroscopy which, in addition to the diamond phase, also confirms the presence of sp${}^{2}$ hybridized carbon bonds. As-grown CVD diamond layers are hydrogen terminated and show p-type conductivity. The effect of the level of hydrogenation on the electrical properties of p-diamond/n-Si heterojunctions has been investigated by temperature dependent current–voltage (J-V/T) characteristics. The obtained results suggest that the energy distribution of interface states at the grain boundary (GB) subjected to hydrogenation becomes shallower, and the hole capture cross-section can be reduced. Hydrogenation can lead to a significant reduction of the GB potential barrier. These results can be interesting from the point of view of hydrogen passivation of GBs in microelectronics.

## 1. Introduction

Diamond is a promising wideband gap semiconductor for high-power devices, owing to its high breakdown electric field, high thermal conductivity, and high bulk carrier mobility. After creating n-type diamond layers using phosphorus (P) doping in 1997 for (111) substrates [1] and in 2005 for (001) substrates [2] research into diamond power devices became popular. Metal semiconductor field effect transistors (MESFETs) based on diamond have shown a high breakdown voltage of over 1.5 kV and high thermal stability up to 573 K [3]. The use of the excellent properties of the diamond for the construction of electronic devices is limited mainly by the inability to obtain shallow acceptors and donors that can facilitate effective conductivity at room temperature [4,5]. The natural elements that can be used to dope diamond are boron and nitrogen due to their similar size to carbon atoms. Nitrogen is a deep donor (1.7 eV), however, due to such high activation energy it proves unsuitable for room temperature performance. It turned out that it is possible to control the electrical properties using nitrogen doping in the case of ultra-nanocrystalline diamond layers [6,7]. Boron is an acceptor with ionization energy equal to 0.37 meV which is still relatively high. The activation energy decreases with increasing boron concentration and tends to zero for boron concentration higher than 10${}^{20}$ cm${}^{-3}$ [8]. At room temperature, only approximately 1% of boron atoms can be activated [9]. It is well known that surfaces of diamonds prepared by chemical vapor deposition (CVD) technique are hydrogen terminated. The hydrogen-terminated surface of diamond exhibits p-type surface conduction [10,11,12,13] in a subsurface region of 10 nm in depth [14]. The surface conduction disappears by oxidation and after that, the surface becomes insulating [15]. Furthermore, Schottky and ohmic metal contacts are easily formed on the same surface by selecting kinds of metals [16,17]. Recently, it has been shown that PDFs materials belong to wide group of semiconductors with a parameter of a positive temperature coefficient of conductivity [18]. Mendes J.C. et al. have been reported that a high conductivity in as-deposited diamond films can be caused through disordered graphitic regions between grains [10] as well as the layers’ hydrogenation has a significant impact on its value too [19]. The aim of the present study was to investigate the electrical characteristics of p-type diamond/n- type Si heterojunction. Its current density-voltage (J-V/T) curves were measured as a function of temperature ranging from 90 K to 300 K. From forward part of characteristics the values for heterojunction parameters such as ideality factor (*n*), Shottky ($\Phi_{B0}$) and grain boundary ($\Phi_{GB}$), barrier heights and saturation current density ($J_{0}$) were extracted. The series resistance ($R_{s}$) were calculated using Cheung’s and modified Norde’s methods [20,21] and it has been correlated with the layer’s hydrogenation as well.

## 2. Materials and Methods

The undoped polycrystalline diamonds have been synthesized by used of the Hot Filament Chemical Vapor Deposition (HF CVD) method. There was applied the mixture of CH${}_{3}$OH/H${}_{2}$ as working gas. The hot filament was made from tungsten wire in the form of coil made from a wire with 0.25 mm in diameter placed about 6 mm above the substrate. The temperature of the filament was measured with an optical pyrometer. The films were synthesized on (111) oriented n-type Si substrate. The substrate temperature was measured with a PtRh10-Pt thermocouple. As a result of doping by As at the level of 10${}^{15}$ cm${}^{-3}$, the Si substrate has a resistivity of 3.5 Ωcm. Parameters of growth processes are presented in Table 1.

Before diamond growth the substrate surfaces were ultrasonically seeded within an ultrasonic cleaner with a suspension of diamond grains in acetone and in a next step they were rinsed by the acetone, the methanol and the distilled water. The synthesis procedure was completed by cooling down the reaction chamber with the sample in a hydrogen atmosphere.

The morphology of obtained diamond layer were studied by a scanning electron microscopy (SEM) (JEOL JSM-820). And the phase purity was characterized with Raman spectroscopy. The Raman spectra were recorded at room temperature in back scattering geometry using Renishaw inVia Raman spectrometer (Renishaw confocal imaging systems). The 488 nm argon laser line was used for excitation. The Raman measurements were made with an accuracy of 1 cm${}^{-1}$. The J-V/T measurement were performed in a configuration of the p-diamond/n-Si heterojunction. The electrical contacts were formed by depositing gold dots of 5 mm in diameter by thermal evaporation on the diamond surface and back of the Si substrate. Characterizations were performed in Oxford Optistat cryostat in range 90–300 K using following instruments: DG1022A Rigol as the power supply generating a rectangular voltage wave with peak to peak amplitude in the range of 4–20 V. The current was registered by 6485 picoammeter Keithley and a voltage drop by 8505A Fluke Digital Multimeter. Each measurement temperature was stabilized by Oxford Mercury controller.

## 3. Results

**Figure 1:**
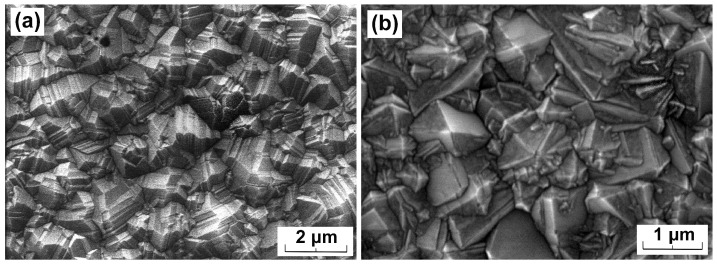
The morphologies of the diamond layers: (**a**) PDF35, (**b**) PDF25.

**Figure 2:**
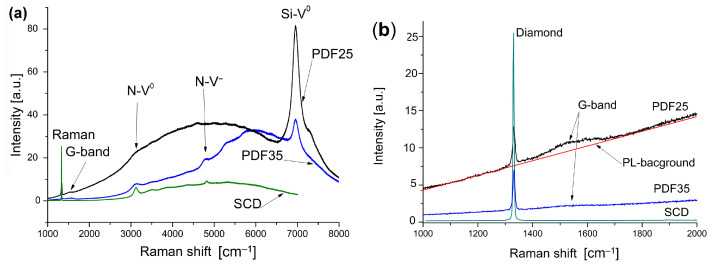
The Raman spectra for both films (**a**) in extended range of wave numbers, and (**b**) in typical range for carbon materials.

The SEM image of the obtained diamond layers i.e., PDF25 and PDF35, are shown in Figure 1. Both samples have thicknesses of 9.5 and 11.5 μm respectively. As one can notice, both diamond layers are polycrystalline in nature, having little different structures. Analyzing the morphologies shown in Figure 1, it can be noticed that the PDF35 sample is characterized by larger microcrystallites, which in turn leads to a lower concentration of grain boundaries. This should be reflected in the Raman spectra, Figure 2.

The diamond’s monocrystal Raman spectrum is characterized by the single line centered at 1332 cm${}^{-1}$ with the full width at half maximum (FWHM) equal to 2–2.5 cm${}^{-1}$ [22]. In the case of the PDFs, in general, the Raman spectrum consists of the sharp diamond’s peak at 1332 cm${}^{-1}$ and a broadband at 1530–1560 cm${}^{-1}$ both superimposed on luminescence background. A broad luminescent background recorded in the extended range of wave numbers of Raman spectra is shown in Figure 2a. For comparison, the Raman spectrum of diamond single crystal (SCD) spectrum provided by Elelement Six is also included. As can be seen, the broadband luminescent background for the single crystal is much weaker than that for the polycrystalline layers. Compared to natural diamond single-crystal, CVD diamond films grown on non-diamond substrates are polycrystalline and generally contain a relatively high concentration of impurities (N, Si see Figure 2a) [23,24], crystal defects and grain boundaries. Due to these defects in Raman spectrum of CVD diamonds, a strong luminescence background is generally observed [25,26,27]. The FWHM of the diamond’s peak is commonly used as a measure of the crystalline quality. The smaller value indicates the better structure of the diamond’s quality. Numerical deconvolution allows to decompose the experimental spectra, shown in Figure 2b, into the part concerning the pure diamond phase and the amorphous carbon phase (C${}_{dia}$) [28] as well. In case of investigated samples, the FWHM is varying from 7.1 cm${}^{-1}$ to 9.9 cm${}^{-1}$ and the sample PDF35 is better quality than the PDF25. The broadband with a maximum at around 1530 cm${}^{-1}$ (the G-band) is ascribed to sp${}^{2}$ hybridized carbon bonds characteristic to the graphite-like structure. Based on Raman studies of many carbon materials, Casiraghi C. et al. [29] have established an empirical formula allowing to estimate the hydrogen content on the basis of the slope of the luminescent background and the integral intensity G-band of the Raman spectrum. Based on this research, we ranked our samples according to their hydrogen content [19], realizing that they were not absolute values. All derived Raman spectra parameters: the diamond’s peak position, the FWHM, the C${}_{dia}$, and the estimation of the hydrogen concentration, (H) [11,19,29] are listed in Table 2.

The analysis of the results presented in Table 2 shows that the PDF35 sample is characterized by better quality of the crystal structure, upper phase purity and higher hydrogen content as well.

**Figure 3:**
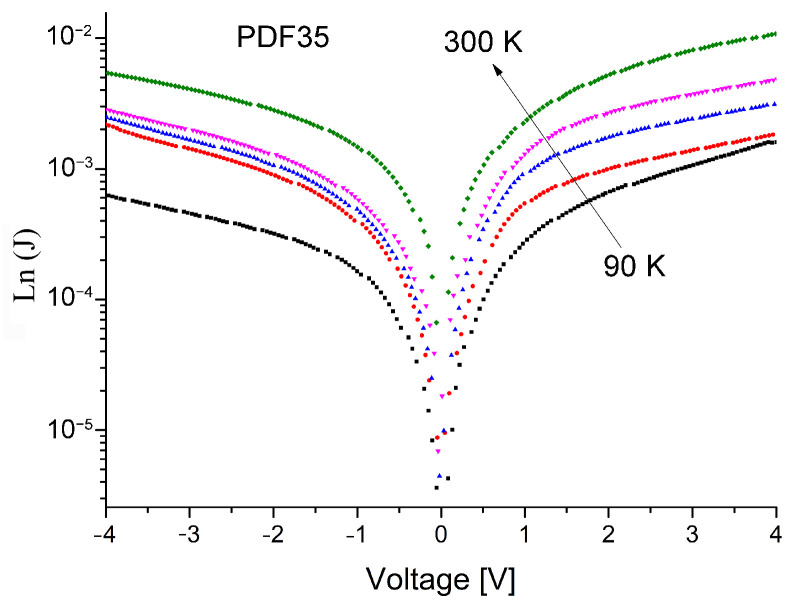
Semilogarithmic plot of the dark J-V characteristic of the present heterojunctions in the voltage range of −4 to +4 V at temperatures from 300 down to 90 K.

The J-V curves for the p-diamond conductivity type grown on n-type Si substrate are displayed in Figure 3 with logarithmic scale as a function of temperature ranging from 300 down to 90 K in the applied bias voltage in the range of −4 to +4 V. All the measurements were carried out in the dark. From particular consideration, at room temperature, the fabricated devices exhibited a light rectifying action similar to conventional p-n abrupt heterojunctions. This is due to the successful formation of a junction between polycrystalline diamond thin film and Si substrate. Similar J-V/T characteristics were also obtained by A.M. Rodrigues, et al. and J.C. Madaleno et al. [30,31]. However, they did not analyze such parameters as the height of the intergrain barriers as well as the electrical resistance of the heterojunction, which is one of the goals of this study.

## 4. Discussion

Using J-V/T characteristics, the values of the main heterojunction’s parameters such as ideality factor (*n*), barrier height ($\phi_{B}$), saturation current density ($J_{0}$) and the series resistance ($R_{s}$) can be calculated using Cheung’s and Norde’s methods [20,21]. For the applied forward bias voltage ≤0.2 V, the forward current ln(J) vs. V characteristic shows a linear dependence. This can be described by means of the thermionic emission (TE) theory as the following relationship [32,33]

(1) $$J=J_{0}\left[exp\left(\frac{qV_{D}}{nkT}\right)-1\right],$$

where: $V_{D}$—applied bias voltage, *J*—the current density, *T*—absolute temperature, *k*—Boltzmann’s constant, *q*—electron charge, *n*—heterojunction ideality factor and $J_{0}$—saturation current density derived from the straight-line intercept of the semilogarithmic forward I–V plot at V = 0. Neglecting 1 ($\frac{qV_{D}}{kT}\gg 1$) in the square bracket of Equation (1) the junction ideality factor *n* can be calculated using the following relation:

(2) $$n=\frac{q}{kT}\frac{d\;V}{d\left(ln\;J\right)}.$$

**Figure 4:**
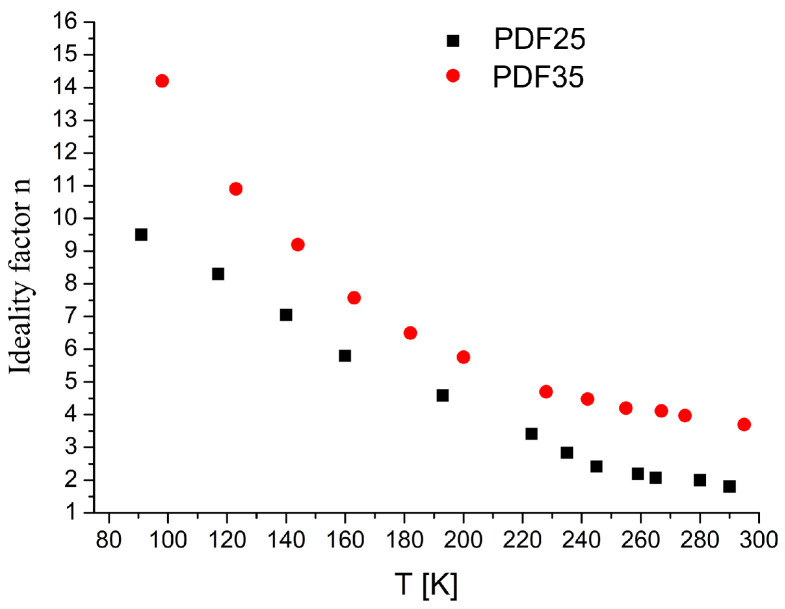
The plot of ideality factor *n* versus temperature for both films.

The temperature dependence of ideality factor *n* is presented in Figure 4.

**Figure 5:**
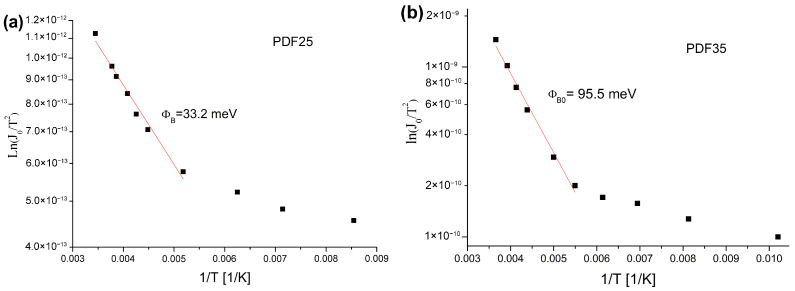
The Richardson plot for samples (**a**) PDF25 and (**b**) PDF35.

Both dependencies display similar behavior, although the *n* for PDF 35 is taking a little higher values. Such large values for the *n* is likely owing to the presence of an flawed contact behavior as well as the result of its inhomogeneity [34]. According to the TE theory, if the value of the *n* is equal to 1 the transportation mechanism of charge carriers across the junction is governed purely by thermal diffusion process. For *n* greater than 1 the generation-recombination and tunneling mechanisms can be considered [35] and series resistance can also effect junction’s ideality factor [36]. It is also reported that the current crowding effect (CCE) has an intense impact on the *n* [37,38]. When the CCE occurs, the distribution of current is not uniform and crowded at the specific region where the separation between p-type material and metal contacts is minimum. The CCE effect can also lead to differentiation of series resistance (see Figure 7b). The saturation current density $J_{0}$ is temperature dependent according to formula [39]:

(3) $$J_{0}=AT^{2}exp\left(\frac{q\phi_{B0}}{kT}\right),$$

where *A*—Richardson constant. The value of the zero-bias Schottky barrier height $\phi_{B0}$ can be calculated from the gradient of so-called Richardson plot shown in Figure 5 [40]. When the surface is hydrogen terminated, the p-type surface conductivity (SC) is induced upon exposure to atmospheric conditions [41,42]. The hydrogenated diamond surface exhibits the negative electron affinity (NEA) which favors SC due to the mechanism of so-called transfer doping [43]. The transfer doping leads to hole accumulation in a subsurface region, at depth in the range from several to even a hundred nanometers. This in turn induces an upward band bending, determining the electrical properties of diamond layers [41]. The band bending at the interface (metal/semiconductor) establishes a built-in potential or Schottky barrier height according to the Schottky-Mott theory [39]. Figure 5 shows that the Schottky barrier is definitely higher for the PDF35 sample, i.e., the sample with a higher degree of hydrogenation. It means that the Schottky barrier height is increased due to the hydrogenation. Similar effect was observed by other researches [44]. The charge carriers must surmount the Schottky barrier to contribute to current flow. Once voltages are applied to the junction, the electrostatics affect the Schottky barrier change. Specificially, its shape and height are altered. The barrier becomes more rounded and the height is reduced with increasing the electric field [45]. The voltage V${}_{D}$ across the diode can be expressed in terms of the total voltage drop V across the series combination of the diode and the resistor. Thus, $V_{D}=V-IR_{s}$, and for V${}_{D}>\frac{3kT}{q}$ Equation (1) will take a form [20]

(4) $$J=J_{0}\left[exp\left(\frac{q\left(V-IR_{s}\right)}{nkT}\right)-1\right].$$

For ideal Schottky diode (*n* = 1) the method for extraction of series resistance $R_{s}$ was proposed by Norde [21] which was then modified by Sato et al. [46] for the case of *n* > 1. Taking Equations (3) and (4) into account and taking them logarithm, we will get:

(5) $$V=IR_{s}+n\phi_{B}+\frac{nkT}{q}ln\left(\frac{J}{AT^{2}}\right).$$

To evaluate the value of barrier height $\phi_{B}$ we define function $H\left(j\right)$ using Equation (5):

(6) $$H\left(J\right)=IR_{s}+n\phi_{B}=V-\frac{nkT}{q}ln\left(\frac{J}{AT^{2}}\right).$$

For ideal Schottky diode (*n* = 1) the method for extraction series resistance $R_{s}$ was proposed by Norde [21] who define Norde function $F\left(V\right)$:

(7) $$F\left(V\right)=\frac{V}{\gamma }-\frac{kT}{q}ln\left(\frac{J}{AT^{2}}\right),$$

where: γ is next integer higher than value of ideality factor *n* (if *n* = 1, the γ = 2). This function was then modified by Cheung et al. [20] proposing the following variation of Norde’s function for *n* > 1:

(8) $$F_{Ch}\left(J\right)=IR_{s}+n\frac{kT}{q}ln\left(\frac{J}{J_{0}}\right).$$

**Figure 6:**
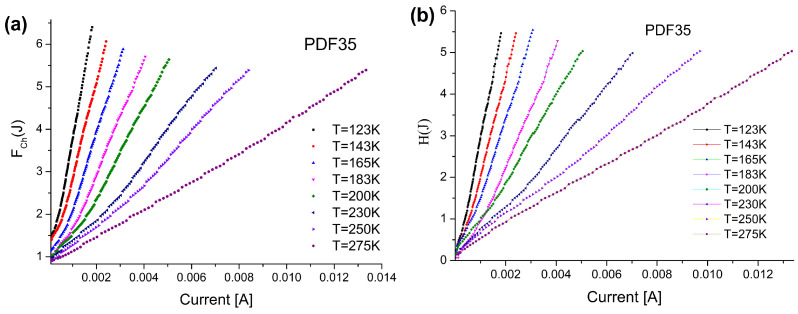
The plot of: (**a**) modified $F_{Ch}\left(J\right)$ and (**b**) $H\left(J\right)$ function vs. current for sample PDF35.

**Figure 7:**
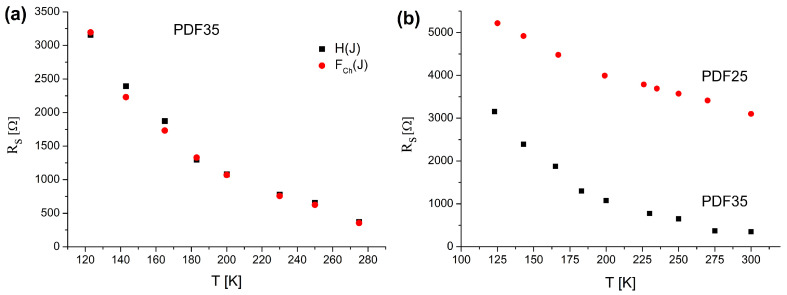
The temperature dependence of $R_{s}$ for PDF35 sample evaluated using both $F_{Ch}\left(J\right)$ and $H\left(J\right)$ functions (**a**), and a tally of the temperature dependence of $R_{s}$ for PDF35 and PDF25 samples (**b**).

The plot of the modified Norde function $F_{Ch}\left(J\right)$ against the current should give a straight line whose slope yields the value of the series resistance $R_{s}$. The plots of $H\left(J\right)$ and modified $F_{Ch}\left(J\right)$ are presented in Figure 6 for PDF35 sample. As it is seen, both functions produce straight lines that slope is temperature dependent. The estimation of the $R_{s}$ value can be made using these two methods independently. As is shown in Figure 7a, both methods give very similar results. Previously indicated (Figure 4), the ideality coefficients *n* for the PDF35 sample were higher, and they could have influenced on the value of the series resistance. This hypothesis is confirmed by the results presented in Figure 7b.

It is clear that for the developed device PDF35 the zero-bais Schottky barrier height $\phi_{B0}$ is higher than for the PDF25 device. And this results in bigger charge accumulation and stronger electrostatic barrier deformation during the current flow. In consequence, lower values of the $R_{s}$ are calculated. In polycrystalline diamond layer, the electrical transport properties are governed not only by the surface hydrogenation but also by carrier trapping at the grain boundary. The entire resistance of these materials should consist of at least two contributions coming from the grain-boundary regions and the crystallite bulk one. However, the current crossing the grain boundary can have two inputs: thermionic emission and tunneling one (field emission). Thermionic emission corresponds to carriers possessing enough high energy to surmount the potential barrier at the grain boundary. In case of lower energy than barrier height, carriers can go through the barrier by tunneling mechanism, if the barrier is enough narrow. If the grains are partially depopulated the electrical conductivity through the grain boundaries is described by formula [47]:

(9) $$\sigma T^{0.5}=Aexp\left(\frac{-\phi_{GB}}{kT}\right).$$

**Figure 8:**
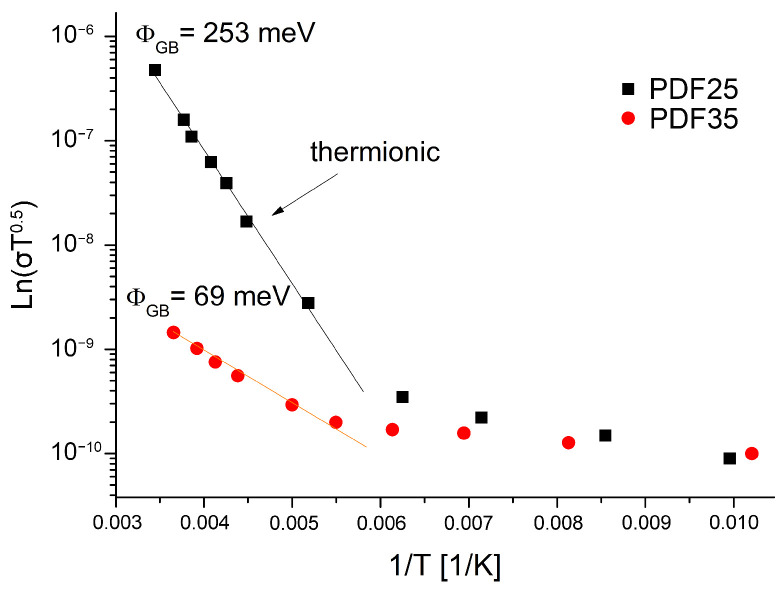
The Arrhenius plot of $ln\left(\sigma T^{0.5}\right)$.

The value of grain boundary barrier height $\phi_{GB}$ can be estimated from linear part of the slope of $ln\left(\sigma T^{0.5}\right)$ vs. 1/T as it is shown in Figure 8.

In this high-temperature region, the flow of charge carriers over the grain boundary barrier is mainly due to a thermionic process [48]. The sample with the lower degree of hydrogenation like PDF25 shows a higher value of the potential barrier height. According to the classical theory of J.Y.W. Seto [47] the height of this barrier in the case of polycrystalline silicon doped with boron (acceptor dopant) decreases with the increase in the doping degree. Several authors [10,49,50] relate the electrical properties of undoped diamond layers mainly to the sp${}^{2}$ carbon phase content. It should be noted, however, that this phase is generally highly hydrogenated. Changing the surface termination from hydrogen to oxygen increases the resistance by several orders [51,52,53]. In our opinion, the reduction of the barrier in the polycrystalline diamond can be explained by assuming that hydrogen, predominantly situated in the grain boundary regions, can play the role of an acceptor impurity like boron in the case of polycrystalline silicon. Hydrogenation of the diamond surface and grain boundaries leads to the mechanism of the so-called doping transfer mechanism [54].

## 5. Conclusions

Diamond films synthesized by a Hot Filament CVD method and their structural properties were characterized using SEM and Raman spectroscopy methods. The p-diamond/n-type Si heterojunction’s parameters, i.e., the values of series resistances $R_{s}$, ideality factors *n*, zero-bias Schottky $\phi_{B0}$ and grain boundary barrier $\phi_{GB}$ heights were calculated from the measured J-V/T characteristics. Higher level of diamond’s surface H-termination leads to higher value of $\phi_{B0}$ caused by higher band bending. The hydrogenation level has an essential influence on the value of series resistance $R_{s}$ and grain boundary barrier height $\phi_{GB}$. Higher level of H-termination results in lowering $R_{s}$ and $\phi_{GB}$ as well. The appropriate adjustment of the hydrogenation level of diamond’s surface can be probably used for tailoring electrical parameters of p-diamond/n-Si heterojunctions.

## Figures and Tables

**Table 1:** Parameters of the diamond growth process.

Films	T${}_{F}$	T${}_{s}$	p	Gas Flow Rate	$\frac{{\mathrm{CH}}_{3}\mathrm{OH}}{H_{2}}$
	[K]	[K]	[hPa]	[sccm]	[vol%]
PDF35	2450 ± 50	1000 ± 50	20 ± 2	100	3
PDF25	2450 ± 50	1000 ± 50	80 ± 2	100	3

T${}_{F}$—filament temperature, T${}_{s}$—substrate temperature.

**Table 2:** Structural parameters evaluated from Raman spectroscopy.

Films	Peak Position	FWHM	C${}_{\mathrm{dia}}$	H
	[cm${}^{-1}$]	[cm${}^{-1}$]	[%]	[at.%]
PDF35	1331.8	7.1	98	17.5
PDF25	1331.6	9.9	93	14.6

## Abbreviations

The following abbreviations are used in this manuscript:

HF CVD	hot filament chemical vapor deposition
J-V/T	current voltage characteristics versus temperature
TE	thermionic emission
RT	room temperature
GB	grain boundary
$\phi_{GB}$	grain boundary’s barrier height
SEM	scanning electron microscopy
FWHW	full width at half maximum
